# Active Ingredients and Action Mechanisms of Yi Guan Jian Decoction in Chronic Hepatitis B Patients with Liver Fibrosis

**DOI:** 10.1155/2019/2408126

**Published:** 2019-09-03

**Authors:** Guangyao Li, Yuan Zhou, Daniel Man-Yuen Sze, Chao Liu, Qianru Zhang, Zihao Wang, Hua Yu, Ging Chan, Zhongdao Wu, Shibing Su, Yuanjia Hu

**Affiliations:** ^1^State Key Laboratory of Quality Research in Chinese Medicine, Institute of Chinese Medical Sciences, University of Macau, Macau SAR, China; ^2^Research Center for Traditional Chinese Medicine Complexity System, Shanghai University of Traditional Chinese Medicine, Shanghai, China; ^3^School of Health and Biomedical Sciences, RMIT University, Melbourne, VIC, Australia; ^4^The Key Laboratory of Tropical Disease Control (Sun Yat-sen University), Ministry of Education, Guangzhou, Guangdong, China; ^5^Department of Parasitology, Zhongshan School of Medicine, Sun Yat-sen University, Guangzhou, Guangdong, China; ^6^School of Pharmacy, Zunyi Medical University, Zunyi, Guizhou, China

## Abstract

**Background and Aim:**

The progression of liver fibrosis in chronic hepatitis B (CHB) patients is currently insufficiently controlled worldwide. The Yi Guan Jian decoction (YGJD) has been widely used in the treatment of liver fibrosis in CHB cases. Although animal studies have reported the antifibrotic effects of the decoction, the active ingredients of the YGJD remain unknown. This study aimed at identifying the potential active ingredients and exploring the mechanisms of action (MOA) of the decoction when treating CHB patients with fibrosis.

**Methods:**

Using data mining techniques and a structural clustering analysis, the potential active ingredients were determined. A network analysis of the differentially expressed genes was conducted to identify the potential targets. Selected compounds were docked to the potential targets for the compound-target interaction simulation. *In vitro* validation, including a cell proliferation assay and Western blot analysis, was conducted to evaluate the prediction results.

**Results:**

In the microarray data, 224 differentially expressed genes related to liver fibrosis were considered to be potential targets. Thirty active ingredients of the YGJD and 15 main targets and relevant pathways were identified. Among them, two active ingredients, methylophiopogonone A and 8-geranyloxypsoralen, were validated as exhibiting antifibrotic effects on hepatic stellate cells.

**Conclusions:**

We identified the potential active ingredients of the YGJD and proposed the possible explanation for the MOA in the treatment of CHB patients with liver fibrosis. Moreover, this study provides a methodological reference for the systematic investigation of the bioactive compounds and related MOA of a traditional Chinese medicine formula in a clinical context.

## 1. Introduction

The hepatitis B virus (HBV) is a major health problem worldwide. Indeed, the World Health Organization has estimated that some 257 million people are infected with the HBV globally, with the virus having been associated with nearly one million deaths in 2015 alone, mainly due to complications such as cirrhosis and liver cancer [1]. In the absence of curative treatment, the most common treatments for the HBV include IFN-*α* and nucleotide analogues, which often lead to severe side effects and drug resistance issues, while having little effect in terms of treating liver fibrosis, cirrhosis, or liver cancer [2].

Due to the issues associated with mainstream treatments, the use of herbal products as a form of complementary medicine has long been widely practiced in Asian countries. In China, over 90% of chronic hepatitis B (CHB) patients received the traditional Chinese medicine (TCM) therapy [3]. Studies have shown various types of TCM to target the HBV [4], liver fibrosis [5], or hepatocellular carcinoma [6]. Further, a meta-analysis of clinical trials found that TCM had an equivalent or better effect than interferon or lamivudine in the treatment of CHB [7]. Moreover, randomized controlled clinical studies have reported the effects of different TCM formulae with regard to improving the clinical parameters of patients with liver fibrosis [8].

As a TCM formula listed in the “Catalogue of Classical TCM Prescriptions” issued by the State Administration of TCM of the People's Republic of China, the Yi Guan Jian decoction (YGJD) is known to have hepatoprotective effects [9]. The YGJD consists of six herbal medicines, namely, Rehmanniae Radix (*Rehmannia glutinosa*), Glehniae Radix (*Glehnia littoralis*), Ophiopogonis Radix (*Ophiopogon japonicus*), Angelica Sinensis Radix (*Angelica sinensis*), Lycii Fructus (*Lycium barbarum*), and Toosendan Fructus (*Melia toosendan*). Three possible mechanisms of action (MOA) have been suggested for the antifibrotic effects of the YGJD: first, the inhibition of hepatic stellate cell (HSC) activation and hepatocytes apoptosis [10]; second, antiangiogenesis via the HIF-1*α*/VEGF signaling pathway [11]; and finally, anti-inflammation [12]. However, the bioactive ingredients of the YGJD remain unknown, while the MOA at the molecular level require further investigation.

In clinical practice involving TCM, the YGJD has been widely used as a basic formula in the treatment of liver-kidney Yin deficiency syndrome (LKYDS) in patients with CHB [13]. LKYDS is mainly seen in CHB patients with severe liver fibrosis [14]. We hypothesized that the beneficial clinical effect of the YGJD in terms of reducing liver fibrosis stems from the oral bioavailable chemical compounds found in the decoction, which are related to the changes in the gene transcription of the blood leukocytes seen in CHB patients with LKYDS. This study hence had two key aims: first, to identify the potential active ingredients of the YGJD with regard to reducing liver fibrosis, and second, to delineate the MOA of the YGJD in relation to the effective treatment of CHB patients with liver fibrosis.

## 2. Materials and Methods

**Figure 1:**
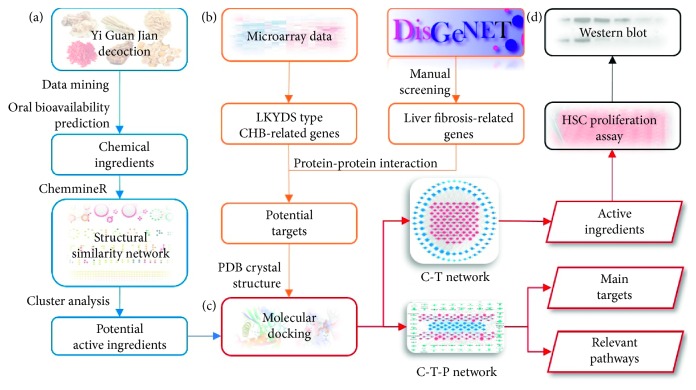
Research roadmap. (a) Collection and screening of the ingredients in the YGJD. (b) Identification of the targets for the YGJD. (c) Network analysis of the active ingredients and the mechanisms of action. (d) Experimental validation.

To achieve the abovementioned research aims, this study gathered relevant bioinformatic data concerning both the YGJD and CHB patients with LKYDS. Further, the study comprehensively employed various methods of analysis, including network pharmacology [15], microarray analysis, molecular docking, and *in vitro* experiments, to analyze the collected data. The research roadmap is shown in Figure 1, while detailed descriptions of the utilized materials and methods are given below.

### 2.1. Compound Collection and Oral Bioavailability Prediction

The relevant chemical information concerning the YGJD was retrieved from four TCM databases in December 2017, including the Traditional Chinese Medicine Systems Pharmacology Database (TCMSP) [16], the Herbal Ingredients Targets Database (HIT) [17], the Psoriasis Database of Traditional Chinese Medicine (PDTCM) [18], and the Potential Target Database of Traditional Chinese Medicine (TCM-PTD). The three-dimensional structures were downloaded from the corresponding database or PubChem.

We used the two parameters proposed by Veber for the preliminary screening of the collected compounds. Veber's filter was used for the oral bioavailability prediction [19], which meant that the number of rotatable bonds of a given compound had to be ≤10, while the topological polar surface area had to be ≤140 Å^2^ [20]. Further, DataWarrior was employed to calculate the molecular properties of the compounds.

### 2.2. Structural Clustering Analysis

The ChemmineR package run in Rstudio was used to compute the fingerprints and the Tanimoto coefficients among the compounds. The obtained cross-similarity matrix was subsequently applied to build the structural similarity network of the compounds with Tanimoto coefficients ≥0.6 [21]. The MCODE plugin in Cytoscape was used to partition the clusters for the network.

### 2.3. Microarray Data Analysis

The microarray data concerning the leukocyte samples obtained from three CHB patients with LKYDS and three healthy donors were provided by the Research Center for TCM Complexity System, Shanghai University of TCM [22]. The differentially expressed genes were identified under the conditions of a fold-change >2 and a *p* value <0.05. The collected genes were then submitted to the STRING database to establish the protein-protein interaction (PPI) network, while the edges with confidence scores greater than 0.7 were retained for further analysis.

The liver fibrosis-related genes were collected from DisGeNET. Using the search term of “Fibrosis, Liver” (UMLS CUI: C0239946), a total of 354 genes were selected from among the results. The CHB with LKYDS genes that exhibited high correlations (a confidence score greater than 0.9 in the STRING database) with the liver fibrosis-related genes were considered to be potential targets for the YGJD. PPI network analysis via pathway enrichment and topological parameter calculation was performed to verify the importance of the potential targets [23].

### 2.4. Molecular Docking

A cross-docking analysis involving sets of compounds and targets was conducted using AutoDock Vina. The crystal structures of the proteins were downloaded from the Research Collaboratory for Structural Bioinformatics Protein Data Bank, and only those X-ray structures with a resolution of less than 3 Å were selected for the analysis ([Supplementary-material supplementary-material-1]). The binding sites were obtained from the original ligand binding locations of the crystal structures, or the reported pockets, or else were predicted by the CASTp 3.0 web server [24]. The gird boxes were adjusted to cover the entire pocket, the majority of which were 22 × 22 × 22 Å^3^. To eliminate any bias caused by the properties of the protein pockets, all the docking scores for each protein were normalized by the median number, as shown in equation (1) [25]. The receiver operating characteristic (ROC) curves between the normalized scores of the active ligands and the ingredients in the YGJD were used to determine the cutoff values [26]:

(1) $$\begin{array}{c} S_{ij}^{\prime }=\frac{\left(S_{ij}-m_{j}\right)}{\sigma_{j}}, \end{array}$$

where *S*_*ij*_ is the original docking score and *S*_*ij*_′ is the normalized score, *i* and *j* are the indexes of the corresponding ligand and protein, respectively, and *m*_*j*_ and *σ*_*j*_ are the median and standard deviation, respectively, of the docking scores of protein *j*.

### 2.5. C-T and C-T-P Network Analysis

Cytoscape was used to visualize the compound-target (C-T) network and the compound-target-pathway (C-T-P) network for the YGJD in the treatment of fibrosis caused by CHB. Two main parameters, namely, the degree centrality (DC) and the betweenness centrality (BC), were used to determine the key nodes in the network [27]. The pathway enrichment of the targets was performed using the STRING database with a false discover rate (FDR) <0.05. In the C-T-P network, the number of shortest paths from all the active ingredients to a biological pathway was exploited to rank the pathways, and the utilized calculation method was as shown in the following equation [28]:

(2) $$\begin{array}{c} P_{m}=\overset{N}{\underset{n=1}{\sum }}k_{mn}{\text{ID}}_{n}, \end{array}$$

where *P*_*m*_ is the number of shortest paths from the active ingredients to pathway *m*, ID_*n*_ is the in-DC of the target *n* in the C-T-P network, *N* represents the number of targets in the network, and *k*_*mn*_ is a dummy variable. Further, *k*_*mn*_=1 when there is a path between the target *m* and the pathway *n*; otherwise, *k*_*mn*_=0.

### 2.6. Cell Proliferation Assay

The LX-2 immortalized human hepatic stellate cell line was purchased from the Type Culture Collection of the Chinese Academy of Sciences (Shanghai, China) [29]. The cells were maintained in Dulbecco's modified Eagle's medium (DMEM, Gibco, USA), which was supplemented with 10% fetal bovine serum (FBS, Gibco, USA), 100 U/mL penicillin, and 100 U/mL streptomycin and then incubated at 37°C in humidified air containing 5% CO_2_.

After being seeded overnight in 96-well plates with DMEM containing 2% FBS, the LX-2 cells were treated with serially diluted compounds for 24 h, 48 h, and 72 h. The cell number was measured by means of a Cell Counting Kit-8 (Dojindo, Kumamoto, Japan) assay. The absorbance was monitored at 450 nm using a microplate reader (PE Envision). Further, the viability percentage was calculated using the following equation:

(3) $$\begin{array}{c} \text{Viability}\%=\frac{T}{C}\times 100\%, \end{array}$$

where *T* and *C* refer to the absorbance of the treatment group and the control group, respectively.

### 2.7. Western Blot Analysis

The cellular content of the collagen I and the *α*-smooth muscle actin (*α*-SMA) in the LX-2 cells was determined by means of Western blot analysis. The LX-2 cells were treated under different conditions with the active ingredients in 5 ng/mL of TGF-*β*1 for 24 h. The lysates of the denatured cells were separated on NuPAGE 4–12% Bis-Tris gel (Thermo Fisher Scientific), and the proteins were transferred to Hybond ECL membranes (GE Healthcare). The membranes were incubated overnight at 4°C with the primary antibodies against collagen type I (Abcam), *α*-SMA (Abcam), and *β*-actin (Sigma) as the endogenous controls, followed by the corresponding secondary peroxidase-coupled antibodies (Sigma). After the chemiluminescence had been enhanced (ECL, Pierce), the digital detection was evaluated using Image Lab Software (ChemiDoc, Bio-Rad).

## 3. Results

### 3.1. Identification of the Representative Compounds of the YGJD

**Figure 2:**
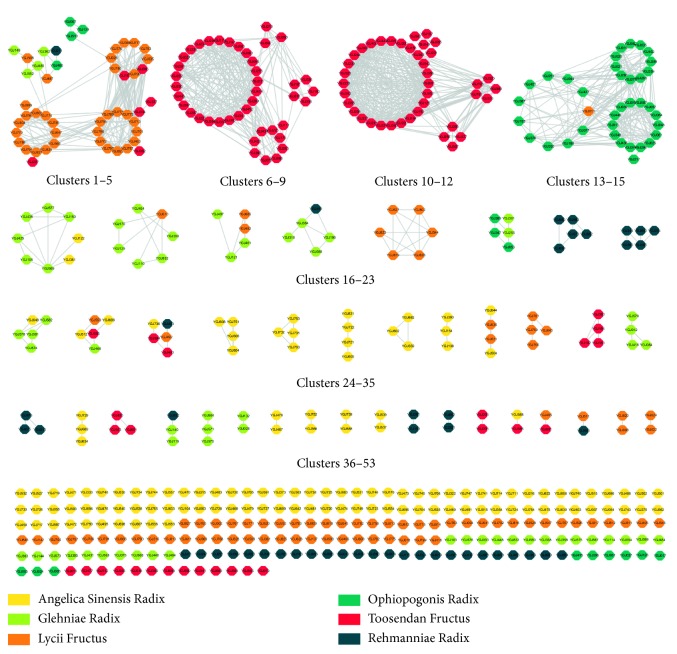
Structural similarity network of the compounds within the YGJD with potential oral bioavailability. The colours of the nodes represent the origin of a compound. The edges in the network represent the connected compounds with a Tanimoto coefficient of no less than 0.6.

A total of 836 compounds contained within the YGJD were acquired from the databases, with 526 being predicted to exhibit better oral bioavailability. A structural similarity network was constructed to identify the representative compounds among the 526 ingredients (Figure 2). The network was divided into 53 clusters. Further investigation of the network showed that the compounds contained within clusters 1–3 were mainly cholestanes from Lycii Fructus, while those within clusters 6–12 were mostly limonoids from Toosendan Fructus, and those within clusters 13–15 were mostly isoflavones from Ophiopogonis Radix.

According to the theory of structure-activity relationship, structurally similar compounds tend to perform similar biological activities [30]. The representative compounds were identified after ranking all the compounds based on their DC and BC scores. The compounds with the top scores in each cluster were selected, giving a total of 57 representative compounds. The representative compounds, in addition to the compounds shown as isolated nodes within the network, were chosen for further docking analysis. In total, 278 compounds ([Supplementary-material supplementary-material-1]) were docked to the selected targets.

### 3.2. The Pool of Potential Targets for the YGJD

**Figure 3:**
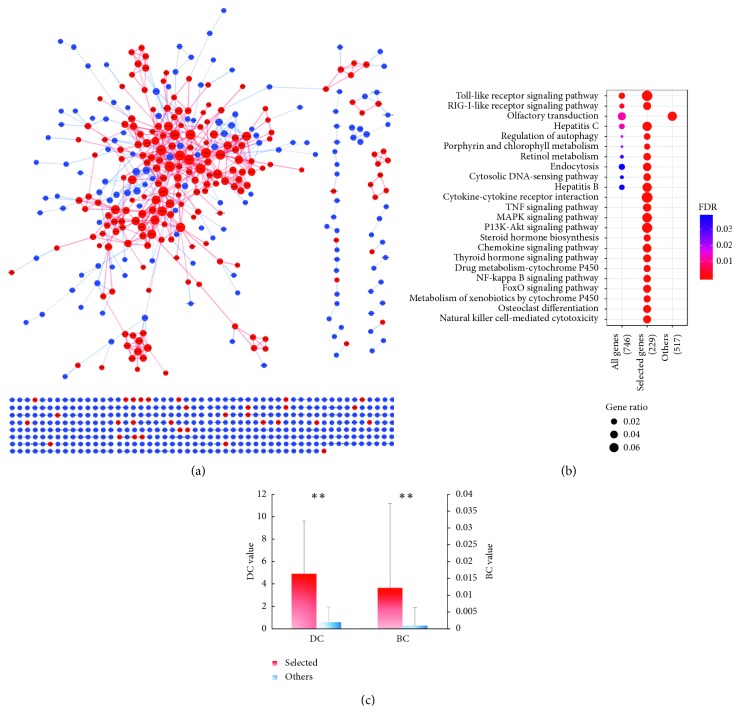
(a) PPI network of the CHB with LKYDS genes. The red nodes represent the genes associated with liver fibrosis, which were selected as potential targets. The sizes of the nodes are proportional to the values of their corresponding DC scores. (b) Results of the pathway enrichment analysis of the CHB with LKYDS genes, the potential targets, and others (genes not selected as potential targets). The sizes of the bubbles represent the gene ratios, which were calculated using the ratio of the pathway-related genes to the total number of gene sets. Only the top 20 enrichment items of the potential targets are shown in the figure. (c) Comparison of the mean values of the DC scores and the BC scores between the selected and the other genes in the network. ^*∗∗*^*P* < 0.01 in the *t*-test for the two independent samples. The standard deviation is shown as the error bar in the chart.

The CHB with LKYDS genes were mapped by means of analyzing the microarray data concerning the CHB with LKYDS patients who had been diagnosed by senior TCM doctors at the Shanghai Longhua Hospital. A PPI network for the differently expressed genes was constructed (Figure 3(a)). In order to obtain the genes highly associated with liver fibrosis and to reduce the potential bias of the microarray data, liver fibrosis-related genes were collected from the DisGeNET database. Some 224 CHB with LKYDS genes that exhibited a high correlation with liver fibrosis were selected as potential targets.

To verify the importance of the selected targets, the different gene sets were subjected to a pathway enrichment analysis (Figure 3(b)) and a topological parameter analysis (Figure 3(c)). After manually deleting those pathways that were apparently unrelated diseases, the potential targets were found to be involved in 64 pathways, while all 740 genes were involved in ten pathways, and those genes not selected as potential targets were only correlated with one pathway. Further, it was found that the selected targets exhibited significantly higher DC and BC scores than the unselected ones. Taken together, the network analysis results indicated the successful exclusion of interfering genes as well as the high correlation between LKYDS and liver fibrosis in CHB patients.

### 3.3. Compound-Target Interaction Simulation Using *In Silico* Molecular Docking

**Figure 4:**
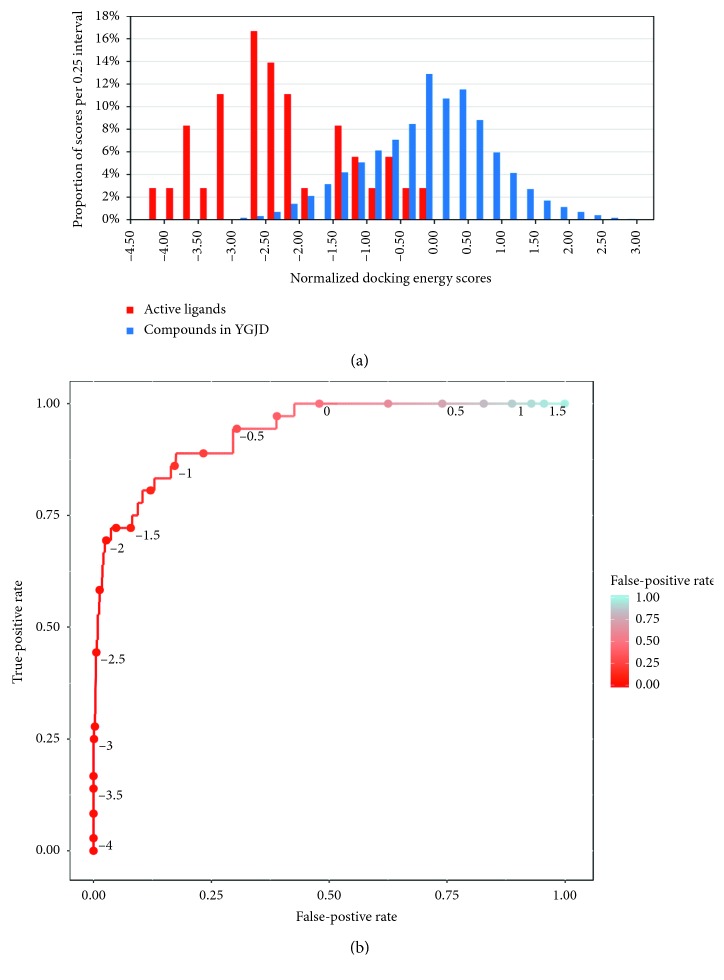
(a) The normalized docking energy score distribution for the active ligands and selected compounds in the YGJD. (b) The ROC curve illustrating the sensitivity/specificity trade-off.

The selected compounds from Figure 2 were docked with the potential targets one to one with AutoDock Vina. The active ligands collected from the protein crystal structures, the DrugBank, or the Therapeutic Target Database were used to determine the cutoff values for the docking scores. The distribution of the normalized docking scores of the active ligands ([Supplementary-material supplementary-material-1]), as well as the selected compounds from the YGJD, is illustrated in Figure 4(a).

The ROC curve presented in Figure 4(b) depicts the sensitivity/specificity trade-off for the threshold values ranging from −4 to 1.5. A cutoff value of −1.75 was selected, with a sensitivity of 0.722 and a specificity of 0.952, and 4.79% of the compound-target interactions were determined to fall within that range. The C-T network was subsequently constructed and visualized based on the predicted compound-target interactions.

### 3.4. Identification of the Active Ingredients in the YGJD That Act on CHB-Related Liver Fibrosis

**Figure 5:**
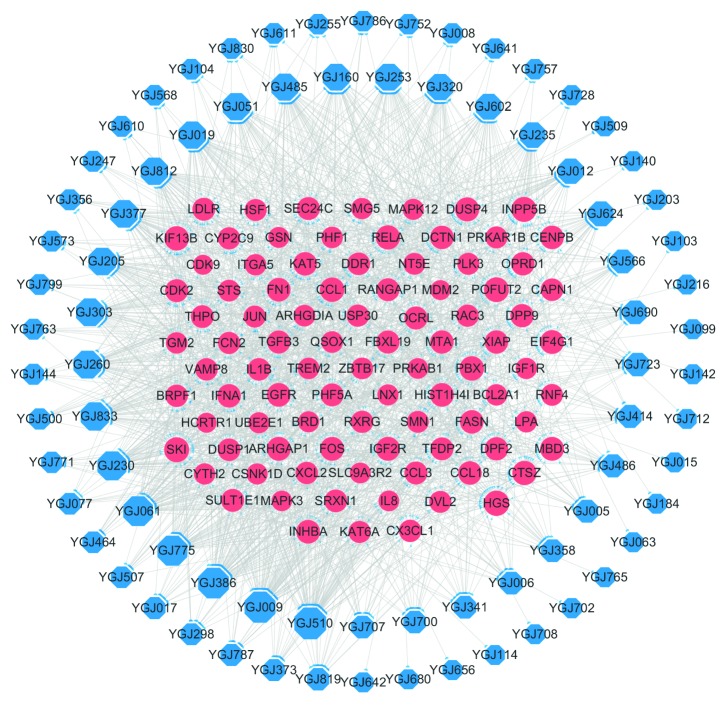
The C-T network based on the results of the molecular docking analysis. The blue octagonal nodes represent the compounds, while the pink elliptical nodes represent the targets.

The C-T network consisted of selected compounds and their respective targets, as visualized in Figure 5. The compounds that exhibited the top 30 out-DC scores were considered to be the active ingredients, and they are listed in Table 1.

### 3.5. MOA for the YGJD When Treating CHB-Related Liver Fibrosis

**Figure 6:**
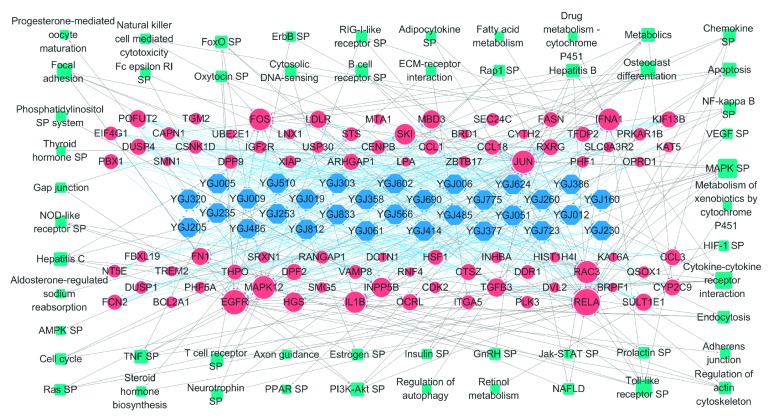
The C-T-P network. The cyan rectangular nodes represent the pathways frequently involved with the potential targets. The sizes of the nodes are proportional to the values of the DC scores. SP: signaling pathway.

The C-T-P network was constructed (Figure 6) to further explore the MOA of the YGJD in the treatment of liver fibrosis caused by CHB. For each active ingredient, the targets that exhibited the top ten highest binding affinities to the ingredients were retained and shown in the graph. The links between the targets and the pathways were drawn based on the results obtained in the pathway enrichment analysis described above.

The BC was used to measure the importance of the targets within the C-T-P network. Those targets with high BC scores were the intermediaries that controlled the flow of interactions within the network. Based on the BC score, the top 15 targets, which are listed in Table 2, were screened as the main targets of YGJD treatment.

Next, based on the C-T-P network, we calculated the shortest paths from the active ingredients to each pathway. By ranking the scores for this indicator, the key biological pathways influenced by the YGJD were identified. The top ten pathways included MAPK signaling pathway, cytokine-cytokine receptor interaction, Toll-like receptor signaling pathway, hepatitis C, endocytosis, PI3K-Akt signaling pathway, hepatitis B, metabolic pathways, chemokine signaling pathway, and osteoclast differentiation. Details concerning these key pathways are provided in [Supplementary-material supplementary-material-1].

### 3.6. Antifibrotic Effects of Methylophiopogonone A and 8-Geranyloxypsoralen on LX-2 Cells

**Figure 7:**
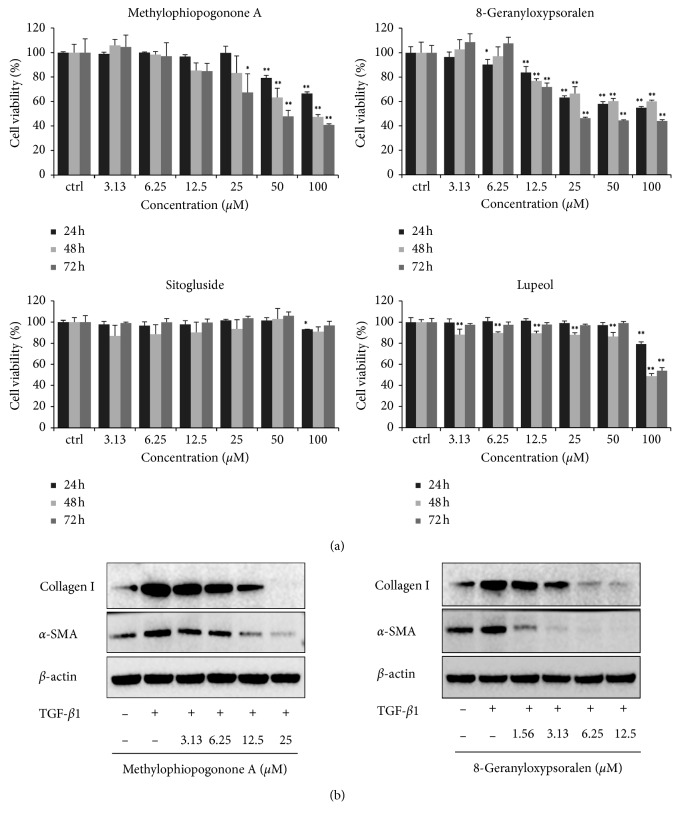
Experimental validation of the antifibrotic effects of the active ingredients in the LX-2 cell line. (a) The viability of the LX-2 cells after treatment with the four active ingredients for 24, 48, and 72 hours. ^*∗*^*P* < 0.05 and ^*∗∗*^*P* < 0.01 in Dunnett's test. (b) The protein levels of collagen I and *α*-SMA induced by TGF-*β*1 (5 ng/ml) in the LX-2 cells after the treatment with methylophiopogonone A and 8-geranyloxypsoralen.

In the 30 active ingredients in Table 1, the 17 representative compounds were selected based on their topological features in Figure 2. The 13 compounds in 17 were considered for the further experimental validation, except for 4 compounds reported to show antifibrotic or hepatoprotective effects in existing literature. In consideration of the availability of these sample compounds, 4 representative compounds in Table 1 were selected for further pharmacological experiments. As shown in Figure 7(a), two ingredients in four, namely, methylophiopogonone A from cluster 15 and 8-geranyloxypsoralen from cluster 35, were found to inhibit the proliferation of the LX-2 cells in a time- and dose-dependent manner. However, there was inadequate evidence to confirm the role of sitogluside and lupeol in inhibiting the proliferation of the LX-2 cells, which indicates the need for further validation of the other potential MOA of the YGJD predicted using our method.

Additionally, two of the main markers of HSC activation, namely, collagen I and *α*-SMA, were determined by means of Western blot analysis after the cells were treated with the compounds for 24 h. As shown in Figure 7(b), the methylophiopogonone A and 8-geranyloxypsoralen inhibited the TGF-*β*1-induced collagen I and *α*-SMA expression.

## 4. Discussion

The YGJD is an herbal formula that is widely used in clinical practice involving TCM for the treatment of CHB patients with LKYDS, a syndrome known to be related to liver fibrosis. Modern pharmacological studies have confirmed the antifibrotic and anti-inflammatory effects of YGJD [31]. In this study, we explored the effects of YGJD on liver fibrosis of CHB by using the LKYDS as a clue to collect microarray data in the specific patient pool of CHB. The microarray data analysis further provided a potential target set in the treatment of liver fibrosis with YGJD from a clinical perspective. The interactions between the compounds of YGJD and the potential targets were revealed by means of molecular docking. Moreover, the method of network pharmacology was used to predict the bioactive ingredients and biological basis of YGJD acting on CHB patients with liver fibrosis.

For the 30 active ingredients predicted by our method, some have been reported to exhibit effects against liver fibrosis. Diosgenin from Ophiopogonis Radix and levistolide A from Angelica Sinensis Radix were observed to inhibit the HSC activation and proliferation [32, 33]. Lupeol, lantadene A, and two trans-ferulic acids from Lycii Fructus, namely, 24-methylenecycloartanol ferulate and campesteryl ferulate, were reported to exhibit hepatoprotective effects in mice with toxically induced liver injuries [34–36]. *N*-*p*-coumaroyltyramine from Ophiopogonis Radix, 8-geranyloxypsoralen from Glehniae Radix, and sitogluside from Rehmanniae Radix were all reported to be involved in anti-inflammatory activities [37–39]. In addition, several limonoids from Toosendan Fructus, such as mesendanin M, trichilinin E, and nimbolinin C, exhibited high out-DC scores in the C-T network. It has previously been reported that limonoids extracted from Meliaceae possess hepatoprotective functions and could hence alleviate liver fibrosis by reducing the TGF-*β*1 and collagen levels [40, 41]. Thus, the limonoids listed in Table 1 might be worthy of further investigation.

Interestingly, 17 of the 30 active ingredients were found to belong to 57 representative compounds in Figure 2, whereas only 13 of the 221 isolated nodes performed well in the docking analysis, which indicated that the highly connected nodes in the compound similarity network had greater opportunity to be biologically active. The compounds with similar structures might represent the driving force within the YGJD, since they could work synergistically to achieve the identified therapeutic effects.

To reveal the MOA of the YGJD in relation to the effective treatment of CHB patients with liver fibrosis, the main targets and the relevant pathways were obtained using the C-T-P network analysis. The findings reported in the literature regarding these principal targets support the suggestion that the YGJD attenuated or even reversed the progression of liver fibrosis among CHB patients using three main approaches. The first approach was the inhibition of the fibrosis-related HSC activation and proliferation. RELA (also known as nuclear factor-*κ*B p65), which has previously been reported to be downregulated in YGJD treatment [42], was found to be a significant factor against the apoptosis of the HSC [43]. Additionally, it has been reported that the inhibition of EGFR could decrease the activation of the HSC [44]. Further, OCRL and INPP5B could regulate HSC proliferation by modulating the AKT signaling pathway [45]. HGS might affect HSC activation via the modulation of both Smad2 and PDGFR [46]. The second approach concerned the fact that some of the main targets were involved in preventing excessive extracellular matrix (ECM) deposition. Fibronectin was part of the ECM [5]. JUN and FOS were reported to influence several genes related to the ECM in the HSC [47]. Moreover, MAPK12 might be involved in regulating *α*1(I) collagen, a key part of the ECM in fibrosis, via the p38 MAPK signaling pathway [48]. The third approach involved regulating the inflammatory response to the HBV by regulating both IL-1B and IFNA1. It has previously been reported that the YGJD could decrease the levels of IL-1B so as to exert anti-inflammatory effects [12]. Further, interferon-alpha encoded by IFNA1 can inhibit the replication of the HBV, thereby leading to the remission of liver diseases [49]. All of the three summarized approaches are consistent with the antifibrotic effects of YGJD reported in the existing literature. This further confirms the accuracy of the target prediction in this study.

The key pathways were also confirmed to be involved in the above-mentioned three approaches accordingly. The MAPK signaling pathway and the PI3K-Akt signaling pathway both played an important role in the entire pathological process of liver fibrosis. Moreover, it was found that the active substances targeting the two pathways could hinder the HSC activation and exert an antifibrotic effect [50, 51]. Cytokine-cytokine receptor interaction, the chemokine signaling pathway, and the Toll-like receptor signaling pathway were all highly related to the immunological process associated with the hepatitis B infection [52]. As for the metabolic pathways, both the retinol metabolism and fatty acid metabolism pathways shown in the C-T-P network were found to be associated with HSC activities as well as with antifibrotic effects [53, 54].

However, a research limitation should be noted. In this study, only two active ingredients were verified to exhibit antifibrotic effects on the HSC by significantly inhibiting the cell proliferation. Further animal experiments and clinical trials on the two ingredients and more potential active ingredients may be performed to explore the scientific nature of YGJD.

## 5. Conclusions

This study predicted 224 potential targets for treating CHB-caused liver fibrosis using microarray data analysis and PPI network analysis and also identified 278 candidates of active ingredients of YGJD through network cluster analysis. The interaction between the compounds and potential targets was revealed by molecular docking analysis and led to a total of 30 active ingredients. Among these active ingredients, methylophiopogonone A and 8-geranyloxypsoralen were found to inhibit HSC proliferation in LX-2 cells *in vitro*. And almost half of the active ingredients have been reported to have antifibrotic or anti-inflammatory effects.

As for the MOA, fifteen genes related to the liver fibrosis caused by the CHB were predicted to be the main targets of YGJD. Literature findings regarding these main targets support YGJD attenuated or even reversed the progression of liver fibrosis among CHB patients by inhibiting fibrosis-related HSC activation and proliferation via RELA, EGFR, OCRL, INPP5B, and HGS, preventing excessive ECM deposition via FN1, JUN, FOS, and MAPK12, and regulating inflammatory response to HBV by regulating both IL-1B and IFNA1. Furthermore, the network analysis revealed the pathways involved in the YGJD treatment, including MAPK signaling, PI3K-Akt signaling, cytokine-cytokine receptor interaction, Toll-like receptor signaling, endocytosis, and metabolism pathway.

In summary, we identified the active ingredients in the YGJD and proposed the possible MOA for the YGJD in the treatment of liver fibrosis among CHB patients. Our study not only provided a direction for the development of new drugs to treat liver fibrosis in CHB patients, but also offered an alternative approach for the systematic investigation of the MOA of a TCM formula by using the YGJD as an example.

## Figures and Tables

**Table 1:** Active ingredients of the YGJD in the treatment of CHB-related liver fibrosis.

ID	Out DC	Compound name	Origin	Molecular formula	Cluster
YGJ510	65	Diosgenin	Ophiopogonis Radix	C_27_H_42_O_3_	3
YGJ009	60	Belladonnine	Lycii Fructus	C_34_H_42_N_2_O_4_	Isolated
YGJ386	59	6-Deacetyloxy-7-deacetylchisocheton	Toosendan Fructus	C_26_H_36_O_4_	Isolated
YGJ775	55	24-Methylenecycloartanol ferulate	Lycii Fructus	C_41_H_60_O_4_	Isolated
YGJ061	54	Methylophiopogonanone A	Ophiopogonis Radix	C_19_H_16_O_6_	15
YGJ230	50	Mesendanin M	Toosendan Fructus	C_30_H_44_O_4_	6
YGJ833	46	Marmesinin	Glehniae Radix	C_20_H_24_O_9_	Isolated
YGJ260	44	Meliasenin X	Toosendan Fructus	C_30_H_48_O_5_	7
YGJ303	43	3,3′Z-6.7′,7.6′-diligustilide	Angelica Sinensis Radix	C_24_H_28_O_4_	Isolated
YGJ205	42	Mesendanin J	Toosendan Fructus	C_28_H_40_O_8_	48
YGJ377	42	Trichilinin E	Toosendan Fructus	C_35_H_42_O_8_	12
YGJ812	41	Lantadene A	Lycii Fructus	C_35_H_54_O_5_	Isolated
YGJ019	40	Ophiopogonone A	Ophiopogonis Radix	C_18_H_14_O_6_	14
YGJ485	39	Lupeol	Glehniae Radix; Lycii Fructus	C_30_H_50_O	2
YGJ051	39	*N*-*p*-coumaroyltyramine	Ophiopogonis Radix	C_18_H_15_NO_4_	Isolated
YGJ253	38	Meliasenin W	Toosendan Fructus	C_30_H_50_O_4_	9
YGJ160	38	Meliasenin P	Toosendan Fructus	C_31_H_50_O_4_	8
YGJ320	35	Nimbolinin C	Toosendan Fructus	C_38_H_46_O_9_	11
YGJ602	32	Levistolide A	Angelica Sinensis Radix	C_24_H_28_O_4_	30
YGJ235	31	Mesendanin U	Toosendan Fructus	C_32_H_50_O_6_	Isolated
YGJ012	30	8-Geranyloxypsoralen	Glehniae Radix	C_21_H_22_O_4_	35
YGJ624	28	Campesteryl ferulate	Lycii Fructus	C_38 _H_56 _O_4_	53
YGJ566	22	24-Methylenecycloartanol	Lycii Fructus	C_31_H_52_O	5
YGJ690	21	12-O-nicotinoylisolineolone	Lycii Fructus	C_27_H_35_NO_6_	Isolated
YGJ723	20	Isotetandrine	Angelica Sinensis Radix	C_38_H_42_N_2_O_6_	Isolated
YGJ486	18	Sitogluside	Rehmanniae Radix; Glehniae Radix; Angelica Sinensis Radix	C_35_H_60_O_6_	51
YGJ414	18	Toosendanic acid A	Toosendan Fructus	C_30_H_48_O_4_	6
YGJ005	17	2′-Hydroxymatteucinol	Ophiopogonis Radix	C_18_H_18_O_6_	Isolated
YGJ006	16	Taurochenodeoxycholic acid	Lycii Fructus	C_26_H_45_NO_6S_	Isolated
YGJ358	16	2-Hydroxy-3-(3-methylbut-2-enyl) furo[3,2 g] chromen-7-one	Glehniae Radix	C_16_H_14_O_4_	Isolated

**Table 2:** Main targets of the YGJD in the treatment of CHB-related liver fibrosis.

Symbol	Entrez ID	Protein name	BC (10^−3^)
RELA	5970	Transcription factor p65, NF-*κ*B subunit	5.28
EGFR	1956	Epidermal growth factor receptor	2.66
RAC3	5881	Ras-related C3 botulinum toxin substrate 3	1.44
FOS	2353	Proto-oncogene c-Fos, AP-1 transcription factor subunit	1.25
IFNA1	3439	Interferon-alpha 1/13	1.05
JUN	3725	Transcription factor AP-1	0.89
MAPK12	6300	Mitogen-activated protein kinase 12	0.61
FN1	2335	Fibronectin	0.59
INPP5B	3633	Type II inositol 1,4,5-trisphosphate 5-phosphatase	0.56
TGFB3	7043	Transforming growth factor-beta 3	0.47
RXRG	6258	Retinoic acid receptor RXR-gamma	0.45
CYP2C9	1559	Cytochrome P450 2C9	0.43
OCRL	4952	Inositol polyphosphate 5-phosphatase OCRL-1	0.36
IL1B	3553	Interleukin-1-beta	0.35
HGS	9146	Hepatocyte growth factor-regulated tyrosine kinase substrate	0.32

## Data Availability

The data that support the findings of this study are available in the Supplementary Materials and openly available in the TCMSP (http://lsp.nwu.edu.cn/tcmsp.php), the HIT (http://lifecenter.sgst.cn/hit/), the PDTCM (http://cadd.gdhtcm.com:2180/PDTCM/), the TCMPTD (http://pharminfo.zju.edu.cn/ptd), the PubChem (https://pubchem.ncbi.nlm.nih.gov/), and the DisGeNET (http://www.disgenet.org/).

## Supplementary Materials

Supplementary Materials Table S1: potential targets for docking analysis. Table S2: selected compounds for docking analysis. Table S3: active ligands as reference for the docking analysis. Table S4: key pathways involved with the YGJD acting on CHB-related liver fibrosis. Table S5: raw data of the docking results.
