# Supplementary material for: Active Ingredients and Action Mechanisms of Yi Guan Jian Decoction in Chronic Hepatitis B Patients with Liver Fibrosis
Source: Evid Based Complement Alternat Med. 2019 Sep 3;2019:2408126. doi: 10.1155/2019/2408126 (PMC6745137; doi:10.1155/2019/2408126)
Supplement: Supplementary Materials — Table S1: potential targets for docking analysis. Table S2: selected compounds for docking analysis. Table S3: active ligands as reference for the docking analysis. Table S4: key pathways involved with the YGJD acting on CHB-related liver fibrosis. Table S5: raw data of the docking results. [file 2408126.f1.zip › 2408126.f1/Supplementary Materials Table S1-S4.docx]

## Supplementary Materials

Table S1: Potential targets for docking analysis

| **Symbol** | **UniProt ID** | **PDB Structure** | **Resolution** | **Symbol** | **UniProt ID** | **PDB Structure** | **Resolution** |
| --- | --- | --- | --- | --- | --- | --- | --- |
| ABL1 | P00519 | 3PYY | 1.85 | INPP5B | P32019 | 4CML | 2.3 |
| ARHGAP1 | Q07960 | 1TX4 | 1.65 | ITGA5 | P08648 | 3VI4 | 2.9 |
| ARHGDIA | P52565 | 2BXW | 2.4 | JUN | P05412 | 5FV8 | 1.99 |
| BCL2A1 | Q16548 | 5WHI | 1.69 | KIF13B | Q9NQT8 | 3GBJ | 2.102 |
| BIRC4 | P98170 | 3HL5 | 1.8 | LDLR | P01130 | 2FCW | 1.26 |
| BRD1 | O95696 | 5FG6 | 1.1 | LNX1 | Q8TBB1 | 3B76 | 1.75 |
| BRPF1 | P55201 | 5FG5 | 1.5 | LPA | P08519 | 4BVC | 1.6 |
| CAPN1 | P07384 | 1ZCM | 2 | MAPK12 | P53778 | 1CM8 | 2.4 |
| CCL1 | P22362 | 4OIK | 2.1 | MAPK3 | P27361 | 4QTB | 1.4 |
| CCL18 | P55774 | 4MHE | 2.1 | MAPK7 | Q13164 | 4ZSL | 2.25 |
| CCL3 | P10147 | 5COR | 2.548 | MBD3 | O95983 | 6CC8 | 1.95 |
| CDK2 | P24941 | 4RJ3 | 1.63 | MDM2 | Q00987 | 4OGN | 1.377 |
| CDK9 | P50750 | 3BLR | 2.8 | MTA1 | Q13330 | 5ICN | 3.3 |
| CENPB | P07199 | 1UFI | 1.65 | MYST3 | Q92794 | 2OZU | 2.3 |
| CSNK1D | P48730 | 4TWC | 1.7 | NT5E | P21589 | 4H2B | 1.7 |
| CTSZ | Q9UBR2 | 1DEU | 1.7 | OCRL | Q01968 | 2QV2 | 2.4 |
| CX3CL1 | P78423 | 4XT1 | 2.886 | OPRD1 | P41143 | 4N6H | 1.8 |
| CXCL2 | P19875 | 5OB5 | 1.65 | PBX1 | P40424 | 1PUF | 1.9 |
| CYP1A2 | P05177 | 2HI4 | 1.95 | PHF1 | O43189 | 5XFP | 2.3 |
| CYP2B6 | P20813 | 3QOA | 2.1 | PHF5A | Q7RTV0 | 5SYB | 1.82 |
| CYP2C9 | P11712 | 5W0C | 2 | PLAU | P00749 | 4FU9 | 1.6 |
| DCTN1 | Q14203 | 2HKN | 1.87 | PLK3 | Q9H4B4 | 4B6L | 1.9 |
| DDR1 | Q08345 | 4BKJ | 1.7 | POFUT2 | Q9Y2G5 | 4AP5 | 3.003 |
| DPF2 | Q92785 | 5VDC | 1.6 | PRKAB1 | Q9Y478 | 4ZHX | 2.99 |
| DPP9 | Q86TI2 | 6EOR | 2.9 | PRKAR1B | P31321 | 4F9K | 2.8 |
| DRD3 | P35462 | 3PBL | 2.89 | PSCD2 | Q99418 | 4JMO | 1.8 |
| DUSP1 | P28562 | 6APX | 2.491 | QSCN6 | O00391 | 3LLK | 2 |
| DUSP4 | Q13115 | 3EZZ | 2.9 | RAC3 | P60763 | 2IC5 | 1.9 |
| DVL2 | O14641 | 3CBX | 1.7 | RANGAP1 | P46060 | 5D2M | 2.4 |
| EGFR | P00533 | 3POZ | 1.5 | RELA | Q04206 | 1NFI | 2.7 |
| EIF4G1 | Q04637 | 1UG3 | 2.24 | RNF4 | P78317 | 2XEU | 1.5 |
| EPAS1 | Q99814 | 3H7W | 1.65 | RXRB | P28702 | 1H9U | 2.7 |
| FASN | P49327 | 4PIV | 2.299 | RXRG | P48443 | 2GL8 | 2.4 |
| FBXL19 | Q6PCT2 | 6ASB | 2.85 | SEC24C | P53992 | 3EH2 | 2.35 |
| FCN2 | Q15485 | 2J0G | 2.85 | SKI | P12755 | 1SBX | 1.65 |
| FN1 | P02751 | 3EJH | 2.1 | SLC9A3R2 | Q15599 | 2OCS | 1.5 |
| FOS | P01100 | 1A02 | 2.7 | SMG5 | Q9UPR3 | 2HWY | 2.75 |
| GRK5 | P34947 | 4TNB | 2.113 | SMN1 | Q16637 | 4QQ6 | 1.75 |
| GSN | P06396 | 5O2Z | 1.7 | SRXN1 | Q9BYN0 | 3CYI | 1.8 |
| HCRTR1 | O43613 | 4ZJC | 2.832 | STS | P08842 | 1P49 | 2.6 |
| HGS | O14964 | 4AVX | 1.68 | SULT1E1 | P49888 | 4JVN | 2.05 |
| HIST1H4I | P62805 | 5JA4 | 2.424 | TFDP2 | Q14188 | 1CF7 | 2.6 |
| HK1 | P19367 | 1CZA | 1.9 | TGFB3 | P10600 | 1TGJ | 2 |
| HSF1 | Q00613 | 5HDN | 1.68 | TGM2 | P21980 | 1KV3 | 2.8 |
| HTATIP | Q92993 | 2OU2 | 2.3 | THPO | P40225 | 1V7M | 2.51 |
| IFNA1 | P01562 | 3UX9 | 2.8 | TKT | P29401 | 4KXX | 1.03 |
| IGF1R | P08069 | 3I81 | 2.08 | TREM2 | Q9NZC2 | 5ELI | 3.0977 |
| IGF2R | P11717 | 2V5O | 2.91 | UBE2E1 | P51965 | 5LBN | 1.42 |
| IL1B | P01584 | 2NVH | 1.53 | USP30 | Q70CQ3 | 5OHK | 2.34 |
| IL8 | P10145 | 5D14 | 1 | VAMP8 | Q9BV40 | 4WY4 | 1.4 |
| INHBA | P08476 | 2ARV | 2 | ZBTB17 | Q13105 | 2Q81 | 2.1 |

Table S2: Selected compounds for docking analysis

| **ID** | **Compound Name** | **Molecular Formula** | **DC** | **BC** | **Oringin** | **Cluster** |
| --- | --- | --- | --- | --- | --- | --- |
| YGJ786 | 31-Norcycloartanol | C_29_H_50_O | 12 | 0.192 | Lycii Fructus | 1 |
| YGJ485 | Lupeol | C_30_H_50_O | 4 | 0.1986 | Lycii Fructus;  Glehniae Radix | 2 |
| YGJ510 | Diosgenin | C_27_H_42_O_3_ | 3 | 0.0799 | Ophiopogonis Radix | 3 |
| YGJ700 | Campesterol | C_28_H_48_O | 20 | 0.1807 | Lycii Fructus | 4 |
| YGJ787 | 31-Norlanost-9(11)-Enol | C_29_H_50_O | 17 | 0.1282 | Lycii Fructus | 5 |
| YGJ566 | 24-Methylenecycloartanol | C_31_H_52_O | 10 | 0.2227 | Lycii Fructus | 5 |
| YGJ414 | Toosendanic Acid A | C_30_H_48_O_4_ | 22 | 0.075 | Toosendan Fructus | 6 |
| YGJ230 | Mesendanin M | C_30_H_44_O_4_ | 12 | 0.0103 | Toosendan Fructus | 6 |
| YGJ260 | Meliasenin X | C_30_H_48_O_5_ | 14 | 0.1472 | Toosendan Fructus | 7 |
| YGJ160 | Meliasenin P | C_31_H_50_O_4_ | 6 | 0.0451 | Toosendan Fructus | 8 |
| YGJ253 | Meliasenin W | C_30_H_50_O_4_ | 15 | 0.104 | Toosendan Fructus | 9 |
| YGJ356 | Trichilinin B | C_35_H_46_O_9_ | 24 | 0.2796 | Toosendan Fructus | 10 |
| YGJ203 | Mesendanin B | C_32_H_44_O_8_ | 5 | 0.2294 | Toosendan Fructus | 10 |
| YGJ320 | Nimbolinin C | C_38_H_46_O_9_ | 17 | 0.018 | Toosendan Fructus | 11 |
| YGJ377 | Trichilinin E | C_35_H_42_O_8_ | 11 | 0.0174 | Toosendan Fructus | 12 |
| YGJ077 | Methylophiopogonanone B | C_19_H_20_O_5_ | 14 | 0.0623 | Ophiopogonis Radix | 13 |
| YGJ019 | Ophiopogonone A | C_18_H_14_O_6_ | 16 | 0.0665 | Ophiopogonis Radix | 14 |
| YGJ061 | Methylophiopogonanone A | C_19_H_16_O_6_ | 19 | 0.083 | Ophiopogonis Radix | 15 |
| YGJ298 | CID53467849 | C_19_H_20_O_6_ | 8 | 0.0788 | Ophiopogonis Radix | 15 |
| YGJ569 | Falcarindiol | C_17_H_24_O_2_ | 7 | 0.6905 | Glehniae Radix | 16 |
| YGJ610 | Scopolin | C_16_H_18_O_9_ | 4 | 0.5333 | Lycii Fructus | 17 |
| YGJ121 | 7-Hydroxycoumarin | C_9_H_6_O_3_ | 3 | 0.5 | Glehniae Radix | 18 |
| YGJ384 | Isopropyl Beta-D-Glucopyranoside | C_9_H_18_O_6_ | 4 | 0.5833 | Glehniae Radix | 19 |
| YGJ819 | Cryptoxanthin | C_40_H_56_O | 5 | 0.0667 | Lycii Fructus | 20 |
| YGJ255 | 6-Beta-Dihydroxyphenethyl Ferulate | C_18_H_18_O_6_ | 3 | 0.25 | Glehniae Radix | 21 |
| YGJ673 | Rehmaglutin B | C_9_H_13_O_5_Cl | 2 | 0.6667 | Rehmanniae Radix | 22 |
| YGJ085 | Sec-Hydroxyaeginetic Acid | C_15_H_24_O_5_ | 3 | 0.8333 | Rehmanniae Radix | 23 |
| YGJ578 | Psoralen | C_11_H_6_O_3_ | 3 | 0.5833 | Glehniae Radix | 24 |
| YGJ466 | Vanillic Acid | C_8_H_8_O_4_ | 2 | 0.6667 | Glehniae Radix | 25 |
| YGJ490 | Cis-Ferulic Acid | C_10_H_10_O_4_ | 2 | 0.6667 | Angelica Sinensis Radix;  Toosendan Fructus | 26 |
| YGJ604 | 3-Butylidenephthalide | C_12_H_12_O_2_ | 3 | 0.6667 | Angelica Sinensis Radix | 27 |
| YGJ753 | 2,4-Xylylaldehyde | C_9_H_10_O | 3 | 0.6667 | Angelica Sinensis Radix | 28 |
| YGJ722 | Senkyunolide | C_12_H_16_O_2_ | 2 | 0.6667 | Angelica Sinensis Radix | 29 |
| YGJ602 | Levistolide A | C_24_H_28_O_4_ | 2 | 0 | Angelica Sinensis Radix | 30 |
| YGJ154 | Senkyunolide-I | C_12_H_16_O_4_ | 2 | 1 | Angelica Sinensis Radix | 31 |
| YGJ636 | Alpha Cadinene | C_15_H_24_ | 3 | 0.6667 | Lycii Fructus | 32 |
| YGJ769 | (1S,4As,6S,8Ar)-1,6-Dimethyldecalin | C_12_H_22_ | 2 | 0.6667 | Lycii Fructus | 33 |
| YGJ184 | Meliaionoside B | C_19_H_36_O_8_ | 3 | 0.1667 | Toosendan Fructus | 34 |
| YGJ012 | 8-Geranyloxypsoralen | C_21_H_22_O_4_ | 3 | 1 | Glehniae Radix | 35 |
| YGJ676 | Rehmaionoside C | C_19_H_32_O_8_ | 2 | 1 | Rehmanniae Radix | 36 |
| YGJ689 | O-Cresol | C_7_H_8_O | 2 | 1 | Angelica Sinensis Radix | 37 |
| YGJ830 | Pinoresinol | C_20_H_22_O_6_ | 2 | 1 | Toosendan Fructus | 38 |
| YGJ140 | 2-Phenylethanol Beta-D-Glucopyranoside | C_14_H_20_O_6_ | 2 | 1 | Glehniae Radix | 39 |
| YGJ571 | Isoimperatorin | C_16_H_14_O_4_ | 2 | 1 | Glehniae Radix | 40 |
| YGJ132 | Threitol | C_4_H_10_O_4_ | 1 | 0 | Glehniae Radix | 41 |
| YGJ467 | Cymol | C_10_H_14_ | 1 | 0 | Angelica Sinensis Radix | 42 |
| YGJ752 | Coniferyl Ferulate | C_20_H_20_O_6_ | 1 | 0 | Angelica Sinensis Radix | 43 |
| YGJ688 | Maruzen M | C_8_H_10_O | 1 | 0 | Angelica Sinensis Radix | 44 |
| YGJ539 | Azelex | C_9_H_16_O_4_ | 1 | 0 | Angelica Sinensis Radix | 45 |
| YGJ281 | Massarigenin C | C_11_H_12_O_5_ | 1 | 0 | Rehmanniae Radix | 46 |
| YGJ351 | Mioporosidegenin | C_12_H_22_O_5_ | 1 | 0 | Rehmanniae Radix | 47 |
| YGJ205 | Mesendanin J | C_28_H_40_O_8_ | 1 | 0 | Toosendan Fructus | 48 |
| YGJ588 | Cis-Thujopsene | C_15_H_24_ | 1 | 0 | Angelica Sinensis Radix | 49 |
| YGJ465 | Protocatechuic Acid | C_7_H_6_O_4_ | 1 | 0 | Lycii Fructus | 50 |
| YGJ486 | Sitogluside | C_35_H_60_O_6_ | 1 | 0 | Angelica Sinensis Radix; Rehmanniae Radix; Glehniae Radix | 51 |
| YGJ820 | Ethyl Anisate | C_10_H_12_O_3_ | 1 | 0 | Lycii Fructus | 52 |
| YGJ624 | Campesteryl Ferulate | C_38_H_56_O_4_ | 1 | 0 | Lycii Fructus | 53 |
| YGJ008 | Dihydropinosylvin | C_14_H_14_O_2_ | 0 | 0 | Angelica Sinensis Radix | Isolated |
| YGJ015 | Brefeldin A | C_16_H_24_O_4_ | 0 | 0 | Angelica Sinensis Radix | Isolated |
| YGJ037 | Beta-Phellandrene | C_10_H_16_ | 0 | 0 | Angelica Sinensis Radix | Isolated |
| YGJ054 | 3,5-Acoradiene | C_15_H_24_ | 0 | 0 | Angelica Sinensis Radix | Isolated |
| YGJ055 | Alaskene | C_15_H_24_ | 0 | 0 | Angelica Sinensis Radix | Isolated |
| YGJ063 | 10-Angeloylbutylphthalide | C_17_H_20_O_4_ | 0 | 0 | Angelica Sinensis Radix | Isolated |
| YGJ104 | Ligustilide Dimer | C_24_H_28_O_4_ | 0 | 0 | Angelica Sinensis Radix | Isolated |
| YGJ176 | Dimethyl Camphorate | C_12_H_20_O_4_ | 0 | 0 | Angelica Sinensis Radix | Isolated |
| YGJ215 | 6Beta,9-Dihydroxy-(+)-Alpha-Pinene | C_10_H_16_O_2_ | 0 | 0 | Angelica Sinensis Radix | Isolated |
| YGJ216 | 9-Hydroxy-(+)-Alpha-Pinene-6Beta-O-D-Glucoside | C_16_H_26_O_7_ | 0 | 0 | Angelica Sinensis Radix | Isolated |
| YGJ275 | 5-Phenylpentan-1,3,4-Triamine | C_11_H_19_N_3_ | 0 | 0 | Angelica Sinensis Radix | Isolated |
| YGJ303 | 3,3'Z-6.7',7.6'-Diligustilide | C_24_H_28_O_4_ | 0 | 0 | Angelica Sinensis Radix | Isolated |
| YGJ322 | Δ2,4-Dihydrophthalic Anhydride | C_8_H_6_O_3_ | 0 | 0 | Angelica Sinensis Radix | Isolated |
| YGJ333 | 2-Methyl-Dodecane-5-One | C_13_H_26_O | 0 | 0 | Angelica Sinensis Radix | Isolated |
| YGJ354 | 1,N-Butylbenzenesulfonamide | C_10_H_15_NO_2_S | 0 | 0 | Angelica Sinensis Radix | Isolated |
| YGJ373 | Sinaspirolide | C_24_H_26_O_4_ | 0 | 0 | Angelica Sinensis Radix | Isolated |
| YGJ403 | Dimethyl Azelate | C_11_H_20_O_4_ | 0 | 0 | Angelica Sinensis Radix | Isolated |
| YGJ459 | Beta-Selinene | C_15_H_24_ | 0 | 0 | Angelica Sinensis Radix | Isolated |
| YGJ460 | Beta-Caryophyllene | C_15_H_24_ | 0 | 0 | Angelica Sinensis Radix | Isolated |
| YGJ468 | Decanal | C_10_H_20_O | 0 | 0 | Angelica Sinensis Radix | Isolated |
| YGJ470 | Beta-Chamigrene | C_15_H_24_ | 0 | 0 | Angelica Sinensis Radix | Isolated |
| YGJ471 | Myrcene | C_10_H_16_ | 0 | 0 | Angelica Sinensis Radix | Isolated |
| YGJ472 | Safrol | C_10_H_10_O_2_ | 0 | 0 | Angelica Sinensis Radix | Isolated |
| YGJ473 | P-Ocimene | C_10_H_16_ | 0 | 0 | Angelica Sinensis Radix | Isolated |
| YGJ474 | Moslene | C_10_H_16_ | 0 | 0 | Angelica Sinensis Radix | Isolated |
| YGJ475 | (+)-Alpha-Terpineol | C_10_H_18_O | 0 | 0 | Angelica Sinensis Radix | Isolated |
| YGJ476 | L-Limonen | C_10_H_16_ | 0 | 0 | Angelica Sinensis Radix | Isolated |
| YGJ479 | Myristicin | C_11_H_12_O_3_ | 0 | 0 | Angelica Sinensis Radix | Isolated |
| YGJ481 | Isodiprene | C_10_H_16_ | 0 | 0 | Angelica Sinensis Radix | Isolated |
| YGJ483 | Succinic Acid | C_4_H_6_O_4_ | 0 | 0 | Angelica Sinensis Radix | Isolated |
| YGJ488 | Galacturonic Acid | C_6_H_10_O_7_ | 0 | 0 | Angelica Sinensis Radix | Isolated |
| YGJ491 | Choline | C_5_H_14_NO | 0 | 0 | Angelica Sinensis Radix | Isolated |
| YGJ495 | Nicotinic Acid | C_6_H_5_NO_2_ | 0 | 0 | Angelica Sinensis Radix | Isolated |
| YGJ501 | Eucarvone | C_10_H_14_O | 0 | 0 | Angelica Sinensis Radix | Isolated |
| YGJ502 | Farnesene | C_15_H_24_ | 0 | 0 | Angelica Sinensis Radix | Isolated |
| YGJ503 | (+)-Alpha-Pinene | C_10_H_16_ | 0 | 0 | Angelica Sinensis Radix | Isolated |
| YGJ515 | Heptanal | C_7_H_14_O | 0 | 0 | Angelica Sinensis Radix | Isolated |
| YGJ527 | Cedrol | C_15_H_26_O | 0 | 0 | Angelica Sinensis Radix | Isolated |
| YGJ530 | Beta-Elemene | C_15_H_24_ | 0 | 0 | Angelica Sinensis Radix | Isolated |
| YGJ531 | Bergamotene | C_15_H_24_ | 0 | 0 | Angelica Sinensis Radix | Isolated |
| YGJ532 | Cuminal | C_10_H_12_O | 0 | 0 | Angelica Sinensis Radix | Isolated |
| YGJ534 | (R)-Linalyl Acetate | C_12_H_20_O_2_ | 0 | 0 | Angelica Sinensis Radix | Isolated |
| YGJ536 | (+)-Verbenone | C_10_H_14_O | 0 | 0 | Angelica Sinensis Radix | Isolated |
| YGJ538 | O-Acetyl-P-Cresol | C_9_H_10_O_2_ | 0 | 0 | Angelica Sinensis Radix | Isolated |
| YGJ540 | (+)-Ledol | C_15_H_26_O | 0 | 0 | Angelica Sinensis Radix | Isolated |
| YGJ553 | Hypnon | C_8_H_8_O | 0 | 0 | Angelica Sinensis Radix | Isolated |
| YGJ557 | Uracil | C_4_H_4_N_2_O_2_ | 0 | 0 | Angelica Sinensis Radix | Isolated |
| YGJ562 | Adenine | C_5_H_5_N_5_ | 0 | 0 | Angelica Sinensis Radix | Isolated |
| YGJ587 | (+)-Cuparene; Cuparene | C_15_H_22_ | 0 | 0 | Angelica Sinensis Radix | Isolated |
| YGJ591 | Hexanoic Acid | C_6_H_12_O_2_ | 0 | 0 | Angelica Sinensis Radix | Isolated |
| YGJ603 | Allocymene | C_10_H_16_ | 0 | 0 | Angelica Sinensis Radix | Isolated |
| YGJ607 | Senkyunolide-D | C_12_H_14_O_4_ | 0 | 0 | Angelica Sinensis Radix | Isolated |
| YGJ609 | (6R)-6-Butylcyclohepta-1,4-Diene | C_11_H_18_ | 0 | 0 | Angelica Sinensis Radix | Isolated |
| YGJ623 | Dictamine | C_12_H_9_NO_2_ | 0 | 0 | Angelica Sinensis Radix | Isolated |
| YGJ630 | 4-Methylphenol | C_7_H_8_O | 0 | 0 | Angelica Sinensis Radix | Isolated |
| YGJ633 | Beta-Bisabolene | C_15_H_24_ | 0 | 0 | Angelica Sinensis Radix | Isolated |
| YGJ635 | Hydron;phenoxide | C_6_H_6_O | 0 | 0 | Angelica Sinensis Radix | Isolated |
| YGJ642 | Naphthalene | C_10_H_8_ | 0 | 0 | Angelica Sinensis Radix | Isolated |
| YGJ694 | Acetovanillin | C_10_H_10_O_4_ | 0 | 0 | Angelica Sinensis Radix | Isolated |
| YGJ695 | Beta-Terpinene | C_10_H_16_ | 0 | 0 | Angelica Sinensis Radix | Isolated |
| YGJ696 | Butal | C_4_H_8_O | 0 | 0 | Angelica Sinensis Radix | Isolated |
| YGJ698 | Mipax | C_10_H_10_O_4_ | 0 | 0 | Angelica Sinensis Radix | Isolated |
| YGJ704 | Ethylbenzaldehyde | C_9_H_10_O | 0 | 0 | Angelica Sinensis Radix | Isolated |
| YGJ705 | Tmhydrop | C_9_H_12_O_2_ | 0 | 0 | Angelica Sinensis Radix | Isolated |
| YGJ708 | Bicycloelemene | C_15_H_24_ | 0 | 0 | Angelica Sinensis Radix | Isolated |
| YGJ711 | 2,4-Dihydroxyacetophenone | C_8_H_8_O_3_ | 0 | 0 | Angelica Sinensis Radix | Isolated |
| YGJ714 | Hydron;phthalate | C_8_H_6_O_4_ | 0 | 0 | Angelica Sinensis Radix | Isolated |
| YGJ717 | (1R,2S,4R)-1-ethyl-1-methyl-2,4-di(propan-2-yl)cyclohexane | C_15_H_30_ | 0 | 0 | Angelica Sinensis Radix | Isolated |
| YGJ719 | Phosphatidylinositol_Qt | C_5_H_9_O_8P_ | 0 | 0 | Angelica Sinensis Radix | Isolated |
| YGJ720 | Phthalic Anhydride | C_8_H_4_O_3_ | 0 | 0 | Angelica Sinensis Radix | Isolated |
| YGJ723 | Isotetandrine | C_38_H_42_N_2_O_6_ | 0 | 0 | Angelica Sinensis Radix | Isolated |
| YGJ724 | Α-Acoradiene | C_15_H_24_ | 0 | 0 | Angelica Sinensis Radix | Isolated |
| YGJ725 | (2S,5R,7R)-2,6,6,8-tetramethyltricyclo[5.3.1.01,5]undec-8-ene | C_15_H_24_ | 0 | 0 | Angelica Sinensis Radix | Isolated |
| YGJ726 | Α-Copaene | C_15_H_24_ | 0 | 0 | Angelica Sinensis Radix | Isolated |
| YGJ727 | 4-Epi-Alpha-Acoradiene | C_15_H_24_ | 0 | 0 | Angelica Sinensis Radix | Isolated |
| YGJ728 | 2,6-Diphenyl-4H-Thiopyran-4-Thione | C_17_H_12_S_2_ | 0 | 0 | Angelica Sinensis Radix | Isolated |
| YGJ730 | 2,4,6-Trimethyl-Octane | C_11_H_24_ | 0 | 0 | Angelica Sinensis Radix | Isolated |
| YGJ733 | 2-Pentanoylbenzoic Acid | C_13_H_16_O_3_ | 0 | 0 | Angelica Sinensis Radix | Isolated |
| YGJ734 | (Z)-2-Hexenyl Hexanoate | C_12_H_22_O_2_ | 0 | 0 | Angelica Sinensis Radix | Isolated |
| YGJ738 | 1,1,5-Trimethyl-2-Formylcyclohexa-2,5-Diene-4-One | C_10_H_12_O_2_ | 0 | 0 | Angelica Sinensis Radix | Isolated |
| YGJ740 | 5-Indolol | C_8_H_7_NO | 0 | 0 | Angelica Sinensis Radix | Isolated |
| YGJ741 | 7,10-Pentadecadiynoic Acid | C_15_H_22_O_2_ | 0 | 0 | Angelica Sinensis Radix | Isolated |
| YGJ743 | Amyl Ketone | C_11_H_22_O | 0 | 0 | Angelica Sinensis Radix | Isolated |
| YGJ744 | Isoamylbenzene | C_11_H_16_ | 0 | 0 | Angelica Sinensis Radix | Isolated |
| YGJ745 | AC1O5TK6 | C_10_H_14_O_4_ | 0 | 0 | Angelica Sinensis Radix | Isolated |
| YGJ746 | Tropone | C_7_H_6_O | 0 | 0 | Angelica Sinensis Radix | Isolated |
| YGJ747 | Aromadendrene | C_15_H_24_ | 0 | 0 | Angelica Sinensis Radix | Isolated |
| YGJ748 | Butyric Acid | C_4_H_8_O_2_ | 0 | 0 | Angelica Sinensis Radix | Isolated |
| YGJ749 | (3S)-Butylphthalide | C_12_H_14_O_2_ | 0 | 0 | Angelica Sinensis Radix | Isolated |
| YGJ750 | D-(+)-Camphoric Acid | C_10_H_16_O_4_ | 0 | 0 | Angelica Sinensis Radix | Isolated |
| YGJ754 | M-Ethylphenol | C_8_H_10_O | 0 | 0 | Angelica Sinensis Radix | Isolated |
| YGJ755 | 1,5,5,6-Tetramethyl-1,3-Cyclohexadiene | C_10_H_16_ | 0 | 0 | Angelica Sinensis Radix | Isolated |
| YGJ002 | Vitamin B5 | C_9_H_17_NO_5_ | 0 | 0 | Lycii Fructus | Isolated |
| YGJ004 | Hyoscyamine | C_17_H_23_NO_3_ | 0 | 0 | Lycii Fructus | Isolated |
| YGJ006 | Taurochenideixycholic Acid | C_26_H_45_NO_6_S | 0 | 0 | Lycii Fructus | Isolated |
| YGJ009 | Belladonnine | C_34_H_42_N_2_O_4_ | 0 | 0 | Lycii Fructus | Isolated |
| YGJ016 | Alanine | C_3_H_7_NO_2_ | 0 | 0 | Lycii Fructus | Isolated |
| YGJ137 | 6-Methyl-3-Hepten-2-One | C_8_H_14_O | 0 | 0 | Lycii Fructus | Isolated |
| YGJ142 | (3S,9R)-Megastigma-6,7-Diene-3,5,9-Triol 9-O-Beta-D-Glucopyranoside | C_19_H_32_O_8_ | 0 | 0 | Lycii Fructus | Isolated |
| YGJ328 | Phenylalanine | C_9_H_11_NO_2_ | 0 | 0 | Lycii Fructus | Isolated |
| YGJ361 | (+/-)-Alpha-Methionine | C_5_H_11_NO_2_S | 0 | 0 | Lycii Fructus | Isolated |
| YGJ405 | 3-Methyldecane | C_11_H_24_ | 0 | 0 | Lycii Fructus | Isolated |
| YGJ416 | Meso-3,5-Dimethyl-Heptan | C_9_H_20_ | 0 | 0 | Lycii Fructus | Isolated |
| YGJ496 | Cystadane | C_5_H_12_NO_2_ | 0 | 0 | Lycii Fructus | Isolated |
| YGJ500 | Physcion | C_16_H_12_O_5_ | 0 | 0 | Lycii Fructus | Isolated |
| YGJ509 | Hypaconitine | C_33_H_45_NO_10_ | 0 | 0 | Lycii Fructus | Isolated |
| YGJ514 | Dibutyl Phthalate | C_16_H_22_O_4_ | 0 | 0 | Lycii Fructus | Isolated |
| YGJ516 | Safranal | C_10_H_14_O | 0 | 0 | Lycii Fructus | Isolated |
| YGJ526 | Paeonol | C_9_H_10_O_3_ | 0 | 0 | Lycii Fructus | Isolated |
| YGJ528 | Farnesylacetone | C_18_H_30_O | 0 | 0 | Lycii Fructus | Isolated |
| YGJ546 | Citric Acid | C_6_H_8_O_7_ | 0 | 0 | Lycii Fructus | Isolated |
| YGJ547 | L-Malic Acid | C_4_H_6_O_5_ | 0 | 0 | Lycii Fructus | Isolated |
| YGJ555 | Vitamin C | C_6_H_8_O_6_ | 0 | 0 | Lycii Fructus | Isolated |
| YGJ611 | Atropine | C_17_H_23_NO_3_ | 0 | 0 | Lycii Fructus | Isolated |
| YGJ612 | 2-Aminoethanesulfonate | C_2_H_7_NO_3_S | 0 | 0 | Lycii Fructus | Isolated |
| YGJ613 | Styrone | C_9_H_10_O | 0 | 0 | Lycii Fructus | Isolated |
| YGJ620 | Beta-Ionone | C_13_H_20_O | 0 | 0 | Lycii Fructus | Isolated |
| YGJ628 | Cumalic Acid | C_6_H_4_O_4_ | 0 | 0 | Lycii Fructus | Isolated |
| YGJ641 | Phenanthren | C_14_H_10_ | 0 | 0 | Lycii Fructus | Isolated |
| YGJ645 | 1,1,6-Trimethyl-2H-Naphthalene | C_13_H_16_ | 0 | 0 | Lycii Fructus | Isolated |
| YGJ646 | 121677 | C_12_H_16_O_3_ | 0 | 0 | Lycii Fructus | Isolated |
| YGJ690 | 12-O-Nicotinoylisolineolone | C_27_H_35_NO_6_ | 0 | 0 | Lycii Fructus | Isolated |
| YGJ702 | Vitamin A | C_20_H_30_O | 0 | 0 | Lycii Fructus | Isolated |
| YGJ706 | Thiamine | C_12_H_17_N_4_OS | 0 | 0 | Lycii Fructus | Isolated |
| YGJ707 | Cyanin | C_29_H_35_N_2_ | 0 | 0 | Lycii Fructus | Isolated |
| YGJ715 | Delta-Cadinol | C_15_H_26_O | 0 | 0 | Lycii Fructus | Isolated |
| YGJ757 | Glycitein | C_16_H_12_O_5_ | 0 | 0 | Lycii Fructus | Isolated |
| YGJ758 | Solavetivone | C_15_H_22_O | 0 | 0 | Lycii Fructus | Isolated |
| YGJ763 | 14B-Pregnane | C_21_H_36_ | 0 | 0 | Lycii Fructus | Isolated |
| YGJ764 | 1,2,3,4,5-Pentamethylcyclopentadiene | C_10_H_16_ | 0 | 0 | Lycii Fructus | Isolated |
| YGJ765 | ZINC15207465 | C_15_H_20_O | 0 | 0 | Lycii Fructus | Isolated |
| YGJ767 | 1,6-Dibromohexane | C_6_H_12Br2_ | 0 | 0 | Lycii Fructus | Isolated |
| YGJ768 | 1,6-Dimethyl-1-Isopropyl-1,2,3,4,4A,7-Hexahydronaphthalene | C_15_H_24_ | 0 | 0 | Lycii Fructus | Isolated |
| YGJ771 | (24R)-4Alpha-Methyl-24-Ethylcholesta-7,25-Dien-3Beta-Ylacetate | C_33_H_54_O_2_ | 0 | 0 | Lycii Fructus | Isolated |
| YGJ775 | 24-Methylenecycloartanol Ferulate | C_41_H_60_O_4_ | 0 | 0 | Lycii Fructus | Isolated |
| YGJ782 | (2-Fluoro-2-Methoxycyclopropyl)Benzene | C_10_H_11_O_F_ | 0 | 0 | Lycii Fructus | Isolated |
| YGJ783 | 2-Methyl-5-Ethyloctane | C_11_H_24_ | 0 | 0 | Lycii Fructus | Isolated |
| YGJ790 | 4-((2Z,5E)-5-Methylhepta-2,5-Dien-2-Yl)Cyclohex-1-Ene | C_14_H_22_ | 0 | 0 | Lycii Fructus | Isolated |
| YGJ791 | 4'-O-Methylnyasol | C_18_H_18_O_2_ | 0 | 0 | Lycii Fructus | Isolated |
| YGJ792 | 1-(2-Hydrazino-4-Methyl-5-Pyrimidinyl)Ethanone | C_7_H_10_N_4_O | 0 | 0 | Lycii Fructus | Isolated |
| YGJ799 | 7-O-Methylluteolin-6-C-Beta-Glucoside_Qt | C_16_H_14_O_7_ | 0 | 0 | Lycii Fructus | Isolated |
| YGJ809 | D-Glutamic Acid | C_5_H_9_NO_4_ | 0 | 0 | Lycii Fructus | Isolated |
| YGJ811 | Ipolamiide_Qt | C_11_H_16_O_6_ | 0 | 0 | Lycii Fructus | Isolated |
| YGJ812 | Lantadene A | C_35_H_52_O_5_ | 0 | 0 | Lycii Fructus | Isolated |
| YGJ813 | Maaliol | C_15_H_24_O_2_ | 0 | 0 | Lycii Fructus | Isolated |
| YGJ816 | Zederone | C_15_H_18_O_3_ | 0 | 0 | Lycii Fructus | Isolated |
| YGJ817 | Aminoethyl Thiosulfite | C_2_H_9_NO_2_S_2_ | 0 | 0 | Lycii Fructus | Isolated |
| YGJ818 | 2-Pyridylamine | C_5_H_6_N_2_ | 0 | 0 | Lycii Fructus | Isolated |
| YGJ825 | Octahydro-4,4,8,8-Tetramethyl-4A,7-Methano-4Ah-Naphth[1,8A-B]Oxirene | C_15_H_24_O | 0 | 0 | Lycii Fructus | Isolated |
| YGJ827 | Trans,Trans-1,6-Dimethyl-Spiro-[4,5]-Decane | C_12_H_22_ | 0 | 0 | Lycii Fructus | Isolated |
| YGJ079 | Loliolide | C_11_H_16_O_3_ | 0 | 0 | Toosendan Fructus | Isolated |
| YGJ235 | Mesendanin U | C_32_H_50_O_6_ | 0 | 0 | Toosendan Fructus | Isolated |
| YGJ341 | Toosendansterol B | C_21_H_34_O_3_ | 0 | 0 | Toosendan Fructus | Isolated |
| YGJ386 | 6-Deacetyloxy-7-Deacetylchisocheton | C_26_H_36_O_4_ | 0 | 0 | Toosendan Fructus | Isolated |
| YGJ477 | (+)-Borneol | C_10_H_18_O | 0 | 0 | Toosendan Fructus | Isolated |
| YGJ518 | 5-Hydroxymethylfurfural | C_6_H_6_O_3_ | 0 | 0 | Toosendan Fructus | Isolated |
| YGJ564 | Cedar Acid | C_9_H_10_O_5_ | 0 | 0 | Toosendan Fructus | Isolated |
| YGJ565 | 4-Hydroxybenzaldehyde | C_7_H_6_O_2_ | 0 | 0 | Toosendan Fructus | Isolated |
| YGJ598 | Torachrysone | C_14_H_14_O_4_ | 0 | 0 | Toosendan Fructus | Isolated |
| YGJ599 | Balanophonin | C_20_H_20_O_6_ | 0 | 0 | Toosendan Fructus | Isolated |
| YGJ600 | Evofolin-B | C_17_H_18_O_6_ | 0 | 0 | Toosendan Fructus | Isolated |
| YGJ616 | Cinnamic Acid | C_9_H_8_O_2_ | 0 | 0 | Toosendan Fructus | Isolated |
| YGJ712 | Cirsiumaldehyde | C_12_H_10_O_5_ | 0 | 0 | Toosendan Fructus | Isolated |
| YGJ075 | (R)-P-Menth-1-Ene-7,8-Diol 8-O-Beta-D-Glucopyranoside | C_16_H_28_O_7_ | 0 | 0 | Glehniae Radix | Isolated |
| YGJ093 | Corchoionoside A | C_19_H_32_O_8_ | 0 | 0 | Glehniae Radix | Isolated |
| YGJ094 | Lasidiol Angelate | C_20_H_32_O_3_ | 0 | 0 | Glehniae Radix | Isolated |
| YGJ099 | 5-Hydroxynorbornyl-O-Beta-D-Glucopyranoside | C_16_H_28_O_7_ | 0 | 0 | Glehniae Radix | Isolated |
| YGJ103 | Adenosine | C_10_H_13_N_5_O_4_ | 0 | 0 | Glehniae Radix | Isolated |
| YGJ114 | Demthyl Furopinnarin | C_16_H_14_O_4_ | 0 | 0 | Glehniae Radix | Isolated |
| YGJ144 | Naphthisoxazol A | C_11_H_9_NO_2_ | 0 | 0 | Glehniae Radix | Isolated |
| YGJ338 | 3,7-Dimethyloct-3(10)-Ene-1,2,6,7-Tetrol | C_10_H_20_O_4_ | 0 | 0 | Glehniae Radix | Isolated |
| YGJ358 | 2-Hydroxy-3-(3-Methylbut-2-Enyl) Furo[3,2-G] Chromen-7-One | C_16_H_14_O_4_ | 0 | 0 | Glehniae Radix | Isolated |
| YGJ363 | Cis-P-Menth-2-Ene-1Α,7,8-Triol | C_10_H_18_O_3_ | 0 | 0 | Glehniae Radix | Isolated |
| YGJ395 | L-Apiitol | C_5_H_12_O_5_ | 0 | 0 | Glehniae Radix | Isolated |
| YGJ431 | Uridine | C_9_H_12_N_2_O_6_ | 0 | 0 | Glehniae Radix | Isolated |
| YGJ440 | Trans-P-Menthane-1Α,2Β,8-Triol | C_10_H_20_O_3_ | 0 | 0 | Glehniae Radix | Isolated |
| YGJ448 | 1-Deoxy-D-Lyxitol | C_5_H_12_O_4_ | 0 | 0 | Glehniae Radix | Isolated |
| YGJ464 | Quercetin | C_15_H_10_O_7_ | 0 | 0 | Glehniae Radix | Isolated |
| YGJ484 | Syrigin | C_17_H_24_O_9_ | 0 | 0 | Glehniae Radix | Isolated |
| YGJ563 | Salicylic Acid | C_7_H_6_O_3_ | 0 | 0 | Glehniae Radix | Isolated |
| YGJ567 | Secoisolariciresinol | C_20_H_26_O_6_ | 0 | 0 | Glehniae Radix | Isolated |
| YGJ568 | Alloisoimperatorin | C_16_H_14_O_4_ | 0 | 0 | Glehniae Radix | Isolated |
| YGJ573 | Marmesin | C_14_H_14_O_4_ | 0 | 0 | Glehniae Radix | Isolated |
| YGJ575 | N-Butyl-Β-D-Fructoufranoside | C_10_H_20_O_6_ | 0 | 0 | Glehniae Radix | Isolated |
| YGJ576 | Nootkatin | C_15_H_20_O_2_ | 0 | 0 | Glehniae Radix | Isolated |
| YGJ580 | Sinapyl Alcohol | C_11_H_14_O_4_ | 0 | 0 | Glehniae Radix | Isolated |
| YGJ833 | Marmesinin | C_20_H_24_O_9_ | 0 | 0 | Glehniae Radix | Isolated |
| YGJ003 | 2-Methoxyhydroquinone | C_7_H_8_O_3_ | 0 | 0 | Ophiopogonis Radix | Isolated |
| YGJ005 | 2'-Hydroxymatteucinol | C_18_H_18_O_6_ | 0 | 0 | Ophiopogonis Radix | Isolated |
| YGJ017 | 4-Demethyl-3,9-Dihydroeucomin | C_16_H_14_O_5_ | 0 | 0 | Ophiopogonis Radix | Isolated |
| YGJ024 | Cyclo(Ile-Leu) | C_12_H_22_N_2_O_2_ | 0 | 0 | Ophiopogonis Radix | Isolated |
| YGJ027 | Beta-Patchoulene | C_15_H_24_ | 0 | 0 | Ophiopogonis Radix | Isolated |
| YGJ051 | N-P-Coumaroyltyramine | C_18_H_15_NO_4_ | 0 | 0 | Ophiopogonis Radix | Isolated |
| YGJ128 | Jasmololone | C_11_H_16_O_2_ | 0 | 0 | Ophiopogonis Radix | Isolated |
| YGJ396 | Ophiopogonoside A | C_21_H_38_O_8_ | 0 | 0 | Ophiopogonis Radix | Isolated |
| YGJ415 | Scopoletin-(3'-Methyl-But-2'-En-1'-Yl)Ether | C_15_H_16_O_4_ | 0 | 0 | Ophiopogonis Radix | Isolated |
| YGJ040 | Norviburtinal | C_9_H_6_O_2_ | 0 | 0 | Rehmanniae Radix | Isolated |
| YGJ115 | Phenylethanoic Acid | C_8_H_8_O_2_ | 0 | 0 | Rehmanniae Radix | Isolated |
| YGJ147 | 2,4-Dimethoxy-2-Methyl-6H-Pyran-3-One | C_8_H_12_O_4_ | 0 | 0 | Rehmanniae Radix | Isolated |
| YGJ163 | 2-(5-Ethenyltetrahydro-5-Methyl-2-Furanyl)-1-(4-Methyl-2-Furanyl)-1-Propanone | C_15_H_20_O_3_ | 0 | 0 | Rehmanniae Radix | Isolated |
| YGJ247 | Aeginetic Acid 5-O-Beta-D-Quinovoside | C_21_H_34_O_8_ | 0 | 0 | Rehmanniae Radix | Isolated |
| YGJ291 | Frehmaglutin E | C_12_H_20_O_3_ | 0 | 0 | Rehmanniae Radix | Isolated |
| YGJ324 | Neoisodihydrocarveol | C_10_H_18_O | 0 | 0 | Rehmanniae Radix | Isolated |
| YGJ369 | Sorbitol | C_6_H_14_O_6_ | 0 | 0 | Rehmanniae Radix | Isolated |
| YGJ399 | Rehmapicrogenin | C_10_H_16_O_3_ | 0 | 0 | Rehmanniae Radix | Isolated |
| YGJ411 | Massarilactone G | C_11_H_12_O_5_ | 0 | 0 | Rehmanniae Radix | Isolated |
| YGJ453 | Glucosamine | C_6_H_13_NO_5_ | 0 | 0 | Rehmanniae Radix | Isolated |
| YGJ489 | Gamma-Aminobutyric Acid | C_4_H_9_NO_2_ | 0 | 0 | Rehmanniae Radix | Isolated |
| YGJ507 | (+)-Neocryptotanshinone | C_19_H_22_O_4_ | 0 | 0 | Rehmanniae Radix | Isolated |
| YGJ560 | Sumiki'S Acid | C_6_H_6_O_4_ | 0 | 0 | Rehmanniae Radix | Isolated |
| YGJ561 | Dihydro-Beta-Ionone | C_13_H_22_O | 0 | 0 | Rehmanniae Radix | Isolated |
| YGJ619 | L-Pyroglutamic Acid | C_5_H_7_NO_3_ | 0 | 0 | Rehmanniae Radix | Isolated |
| YGJ654 | Melittoside_Qt | C_9_H_12_O_5_ | 0 | 0 | Rehmanniae Radix | Isolated |
| YGJ656 | Geniposide_Qt | C_11_H_14_O_5_ | 0 | 0 | Rehmanniae Radix | Isolated |
| YGJ658 | Jiofuran | C_9_H_12_O_4_ | 0 | 0 | Rehmanniae Radix | Isolated |
| YGJ661 | Jioglutin D | C_11_H_18_O_6_ | 0 | 0 | Rehmanniae Radix | Isolated |
| YGJ663 | Jioglutolide | C_9_H_14_O_4_ | 0 | 0 | Rehmanniae Radix | Isolated |
| YGJ666 | Jioglutoside B_Qt | C_11_H_14_O_4_ | 0 | 0 | Rehmanniae Radix | Isolated |
| YGJ674 | (3As,4R,6As)-4-Hydroxy-6,6A-Dimethylol-3A,4-Dihydro-3H-Cyclopenta[D]Furan-2-One | C_9_H_12_O_5_ | 0 | 0 | Rehmanniae Radix | Isolated |
| YGJ680 | Rehmapicroside | C_16_H_26_O_8_ | 0 | 0 | Rehmanniae Radix | Isolated |
| YGJ760 | Ajugoside_Qt | C_11_H_16_O_5_ | 0 | 0 | Rehmanniae Radix | Isolated |

Table S3: Active ligands as reference of the docking analysis

| **Active ligand** | **Ligand PDB ID** | **Protein PDB Structure** | **Protein Symbol** | **Docking score** | **Normalized Score** | **Ligand source** |
| --- | --- | --- | --- | --- | --- | --- |
| Imatinib | STI | 3PYY | ABL1 | -13.3 | -2.66897 | Crystal structure |
| PFI-4 | 5XF | 5FG5 | BRPF1 | -7.3 | -1.39868 | Crystal structure |
| CHEMBL3354189 | 3QS | 4RJ3 | CDK2 | -10.1 | -2.26554 | Crystal structure |
| Flavopiridol | CPB | 3BLR | CDK9 | -9.5 | -1.48103 | Crystal structure |
| CHEMBL3337854 | 37J | 4TWC | CSNK1D | -10 | -2.04197 | Crystal structure |
| 2-PHENYL-4H-BENZO[H]CHROMEN-4-ONE | BHF | 2HI4 | CYP1A2 | -15.7 | -1.03365 | Crystal structure |
| 4-benzylpyridine | 3QO | 3QOA | CYP2B6 | -8 | -0.15108 | Crystal structure |
| CHEMBL2440218 | JAF | 4JMO | CYTH2 | -6.9 | -0.99167 | Crystal structure |
| Imatinib | STI | 4BKJ | DDR1 | -12.7 | -3.69494 | Crystal structure |
| 1G244 | 9XH | 6EOR | DPP9 | -9.8 | -2.60782 | Crystal structure |
| Eticlopride | ETQ | 3PBL | DRD3 | -8.1 | -0.53079 | Crystal structure |
| Okadaicacid | NA | 6APX | DUSP1 | -8.1 | -3.96034 | TTD |
| Okadaicacid | NA | 3EZZ | DUSP4 | -7.5 | -3.28225 | [http:\\dx.doi.org\10.5012\bkcs.2014.35.9.2655](http://dx.doi.org/10.5012/bkcs.2014.35.9.2655) |
| TAK-285 | 03P | 3POZ | EGFR | -10.5 | -2.66695 | Crystal structure |
| SCHEMBL15717116 | 018 | 3H7W | EPAS1 | -8.4 | -0.25239 | Crystal structure |
| GSK2194069 | 2W4 | 4PIV | FASN | -12.5 | -3.14942 | Crystal structure |
| SB-674042 | 4OT | 4ZJC | HCRTR1 | -10.6 | -1.33479 | Crystal structure |
| BMS-754807 | EBI | 3I81 | IGF1R | -10.2 | -3.19299 | Crystal structure |
| Talmapimod | NA | 2NVH | IL1B | -8.1 | -3.17354 | DRUGBANK |
| Dioscin | NA | 3VI4 | ITGA5 | -6.9 | -2.59917 | <https://doi.org/10.1016/j.fct.2017.07.014> |
| Acetyl coenzyme *A | ACO | 2OU2 | KAT5 | -9.1 | -2.34964 | Crystal structure |
| Adenosine 5'-diphosphate | ADP | 3GBJ | KIF13B | -8.3 | -2.43961 | Crystal structure |
| CHEMBL3590107 | 38Z | 4QTB | MAPK3 | -14.5 | -4.15168 | Crystal structure |
| CHEMBL3236356 | 2U5 | 4OGN | MDM2 | -9.9 | -2.078 | Crystal structure |
| Baicalin | 0XE | 4H2B | NT5E | -10 | -2.12092 | Crystal structure |
| Naltrindole | EJ4 | 4N6H | OPRD1 | -11.7 | -3.53713 | Crystal structure |
| 6-(N-Phenylcarbamyl)-2-Naphthalenecarboxamidine | 675 | 4FU9 | PLAU | -8 | -1.21161 | Crystal structure |
| BNAAGNVFCJFJIF-AUSIDOKSSA-N | 9ZP | 4B6L | PLK3 | -10.7 | -2.35113 | Crystal structure |
| A-769662 | C1V | 4ZHX | PRKAB1 | -6.4 | -2.54697 | Crystal structure |
| Flavin adenine dinucleotide | FAD | 3LLK | QSOX1 | -12.6 | -3.70582 | Crystal structure |
| LG-100268 | LG2 | 1H9U | RXRB | -13.1 | -2.25267 | Crystal structure |
| GYFRQCMDLBNZSF-UHFFFAOYSA-N | NA | 4QQ6 | SMN1 | -7.3 | -1.8616 | DRUGBANK |
| Adenosine triphosphate | ATP | 3CYI | SRXN1 | -6.9 | -2.73944 | Crystal structure |
| 2,6-Dibromo-3-(2,4-dibromophenoxy)phenol | YUG | 4JVN | SULT1E1 | -7.5 | -0.53059 | Crystal structure |
| Guanosine 5'-diphosphate | GDP | 1KV3 | TGM2 | -7.1 | -2.13425 | Crystal structure |
| SCHEMBL12105442 | 9JZ | 3HL5 | XIAP | -7.6 | -3.00016 | Crystal structure |

Table S4: Key pathways involved with the YGJD acting on CHB-related liver fibrosis

| **ID** | **Pathway** | **Proteins linking to the pathway** | **P_m_** |
| --- | --- | --- | --- |
| 4010 | MAPK signaling pathway | DUSP1, DUSP4, EGFR, FOS, IL1B, JUN, MAPK12, RAC3, RELA, TGFB3 | 38 |
| 4060 | Cytokine-cytokine receptor interaction | CCL1, CCL18, CCL3, EGFR, IFNA1, IL1B, INHBA, TGFB3 | 28 |
| 4620 | Toll-like receptor signaling pathway | CCL3, FOS, IFNA1, IL1B, JUN, MAPK12, RELA | 25 |
| 5160 | Hepatitis C | EGFR, IFNA1, LDLR, MAPK12, RELA | 24 |
| 4144 | Endocytosis | EGFR, HGS, LDLR, TGFB3 | 23 |
| 4151 | PI3K-Akt signaling pathway | EGFR, FN1, IFNA1, ITGA5, RELA | 22 |
| 5161 | Hepatitis B | FOS, IFNA1, JUN, RELA, TGFB3 | 21 |
| 1100 | Metabolic pathways | CYP2C9, FASN, INPP5B, NT5E, OCRL | 21 |
| 4062 | Chemokine signaling pathway | CCL1, CCL18, CCL3, RELA | 20 |
| 4380 | Osteoclast differentiation | FOS, IL1B, JUN, MAPK12, RELA, TREM2 | 18 |
